# Person-Centered Exploration of Neonatal Intensive Care Unit Stressors and Social Support in Parenting Very Preterm Infants: A Cross-Sectional Study on Risks and Resources in Italy and Portugal

**DOI:** 10.3390/children13060832

**Published:** 2026-06-18

**Authors:** Federica Vallone, Carmine Vincenzo Lambiase, Mariana Amorim, Susana Silva, Milton Severo, Francesco Raimondi, Maria Clelia Zurlo

**Affiliations:** 1 Dynamic Psychology Laboratory, Department of Humanities, University of Naples Federico II, 80133 Naples, Italy; federica.vallone@unina.it (F.V.); carminevincenzo.lambiase@unina.it (C.V.L.); 2 EPIUnit/ITR, Institute of Public Health, University of Porto, 4050-600 Porto, Portugal; mariana.amorim@ispup.up.pt (M.A.); milton@ispup.up.pt (M.S.); 3 Institute of Social Sciences, University of Minho, 4710-057 Braga, Portugal; susilva@ics.uminho.pt; 4 Division of Neonatology, Department of Translational Medical Sciences, University of Naples Federico II, 80131 Naples, Italy; raimondi@unina.it

**Keywords:** cross-country comparison, fathers, mothers, multigroup latent class analysis, neonatal intensive care units, parenting, person-centered approach, stressors, sources of support, very preterm infants

## Abstract

**Highlights:**

**What are the main findings?**

- The study provides cross-country evidence to advance the understanding of the nuanced experiences of parents of VPT infants in NICUs.
- The study offers three statistically valid, cross-country classes that group parents of VPT infants in NICUs according to perceived risks (NICU stressors) and resources (sources of social support).

**What is the implication of the main finding?**

- Findings can be used to inform the definition of evidence-based, tailored interventions aimed at providing parents with the necessary resources to adjust to this extremely demanding experience.

**Abstract:**

Objective: Based on the Person-Centered Approach, this study targeted parents of very preterm (VPT) infants in Neonatal Intensive Care Units (NICUs) from Italy and Portugal. The primary aim was to classify parents by identifying latent classes of perceived risks (NICU stressors) and resources (sources of social support). Potential specificities in class membership according to Country of Belonging and sociodemographic factors were also investigated. Methods: Overall, 303 parents (92 Italian; 211 Portuguese) completed a survey including sociodemographic factors, Parental-Stressor-Scale-NICU, and Multidimensional-Scale-of-Perceived-Social-Support. Data were analyzed by multigroup latent class analysis and multinomial logistic regression. Results: Three statistically valid and cross-country classes were identified and labelled as Class 1, *Adjusted*/*Beneficial-and-Supported-System*, Class 2, *Stressed-and-Supported-System*, and Class 3, *Parental-Role-Alteration-with-Family-Supported-System*. Portuguese parents were mainly grouped in Classes 1 and 2, while Italian parents were in Class 3. Men were less likely to belong to Classes 2 and 3, while older parents having another child were more likely to belong to Class 3. Conclusions: The experience of parents of VPT infants in NICUs is inherently challenging, yet identifying specific risk profiles featured by the unique nuances of stressors and sources of support while accounting for further factors (Country of Belonging, Gender, Age, Having another child) can foster the customization of interventions aimed at providing parents with the necessary resources for adjusting to this extremely demanding experience.

## 1. Introduction

Research has widely demonstrated the challenges of parenting [1]. Therefore, providing effective support systems and interventions that foster caregiving roles has been prioritized in international agendas [2,3]. However, this is even more challenging when the process of adjusting to parenthood and caring for one’s newborn is complicated by preterm birth and subsequent transfer to the Neonatal Intensive Care Unit (NICU) [4,5]. In this environment, the *potential* of this fragile new life is indeed “suspended”, and—despite all efforts—the newborn may not survive [6,7,8].

Although infant mortality rates have decreased substantially in recent decades, preterm birth remains the leading cause of death among children under five years of age worldwide [9,10]. Furthermore, very preterm infants (VPT; born at <32 weeks of gestation) are at a higher risk of mortality than other preterm infants [11]. In such cases, parents must deal with not only fears over their child’s survival, but also with additional—often long-lasting—challenges to their parenting and care opportunities [12].

From this perspective, based on the *Parental Preterm Distress Model* [13], research in the field has extensively highlighted that parents of VPT infants may report overwhelming levels of stress, burden, and psychopathological symptoms [5,14,15]. However, the literature has also identified three potential major sources of stress for NICU parents: 1. the *Sights and Sounds stressor* (SISO); 2. the *Infant and Behaviour Appearance stressor* (IBA); 3. the *Parental Role Alteration stressor* (PRA) [16]. Specifically, the NICU environment itself is the first challenging element to consider, as it exposes parents to persistent visual and auditory stimuli from medical devices and alarms, as well as to the sight of their baby surrounded by other critically ill infants (SISO). Within this environment, viewing one’s own infant as unhealthy and extremely fragile, surrounded by tubes and intravenous lines and subjected to painful and invasive procedures (IBA), alongside with experiencing frustration due to an inability to perform the expected parenting role adequately (PRA), are key risk factors that have been highlighted as adversely affecting parental adjustment to hospitalization [17,18], the health of parents and infants [4], and the parent–infant relationship and closeness [19].

However, research in the field has also suggested the need to account for the role of key resources that can effectively help parents deal with this emotionally demanding experience [19,20,21,22]. Above all, *social support* has been shown to play a pivotal protective role both in the short term (during the hospitalization and the postpartum period) and in the long term, in particular after the discharge [23,24,25,26]. Specifically, within the NICU context, research on social support systems has underlined the importance of significant others (e.g., peers, spouses [27,28,29]) and family and friends [30,31,32] in providing both emotional and practical help (e.g., caring for other children, providing financial aid).

Notwithstanding the clear identification of specific sources of stress featuring, parents’ NICU experience, along with social resources potentially alleviating their burden, there is a lack of research examining the nuance of their unique experience. Parents, indeed, can perceive specific sources of stress and social support with varying intensities and, accordingly, they can adjust differently to the NICU experience. Shedding light on this complexity could, in turn, help to better understand the different needs that must be considered when defining the appropriate intervention.

In line with this, over the last decades, a growing number of studies adopted a Person-Centered Approach [33,34,35,36]. This is a conceptual framework including an array of statistical methods (e.g., Latent-Class-Analysis, Cluster Analyses) aiming to identify subgroups of subjects with similar profiles [37,38,39] to develop customized interventions. By focusing on individuals instead of study variables, the person-centered approach allows researchers and practitioners to achieve a more complex understanding of real-life experiences [40]. For example, this approach is able to capture how people can perceive high burden while simultaneously possessing different resources to deal with it.

Within the NICU context, a person-centered approach has been used to prospectively detect the path of maternal psychological distress profiles during NICU hospitalization and their consequences on the mother’s psychological health and on child outcomes after discharge [41,42], as well as to retrospectively evaluate the array of parental experiences of care during NICU hospitalization [43].

However, to the best of our knowledge, the person-centered approach has never been applied to evaluate the complex interplay between risks and resources potentially characterizing the experience of parents in the NICU, necessitating further studies in this research direction. The adoption of a person-centered approach is—indeed—of particular interest for NICU research, mainly when not only risks but also sources of support are addressed. In fact, the person-centered approach fully aligns with the Family-Centered Care Model (FCC Model), which is considered the gold standard in NICUs [44,45,46]. Specifically, the FCC is a model “where care is planned around the whole family” [47]. It is driven by the idea of providing the family with adequate and tailored support. This, however, can only be achieved by assessing each family’s unique needs to provide appropriate and adequate support.

From this perspective, research in the field still highlights several barriers to the effective implementation of FCC interventions within NICUs, mainly linked to the lack of evidence-based interventions, thus underpinned by a careful and tailored assessment of specific sources of stress, resources, and needs reported by the parents [44,47].

Moreover, another aspect hindering the effective implementation of FCC is represented by the presence of differences and specificities in NICU care protocols, parental access to infants, possibility for involvement (visiting policies) of members of the parental social support system across European countries [48,49]. This calls for the development of further comparative studies across different settings and countries [19,44] in order to implement practices that align with the European Standards of Care for Newborn Health (ESCNH) while accounting for country specificities.

### The Present Study

Responding to all the abovementioned research needs, and considering the international priority to provide parents of very preterm (VPT) infants in NICUs with tailored and effective support interventions, this study adopted a person-centered approach to explore and compare the experiences of parents of VPT infants in NICUs from two European countries, namely Italy and Portugal. The primary objective was to classify parents by identifying latent classes of perceived risks (NICU stressors, i.e., the *Sights and Sounds stressor* [SISO]; the *Infant and Behaviour Appearance stressor* [IBA]; and the *Parental Role Alteration stressor* [PRA]) and resources (sources of social support, i.e., family, friends and significant others). Moreover, to provide more accurate evidence to customize interventions, potential specificities in class membership according to Country of Belonging and to sociodemographic factors were also investigated.

The selection of a defined set of sociodemographic characteristics was underpinned by literature in the field, which highlighted specific factors as potentially playing a role in influencing the NICU experience, namely: Gender (e.g., being mother or father [50]); Age, Education level, Occupational status [51,52]; Distance between home and the NICU [53]; and Previous Pregnancies (having another/other children or previous pregnancy loss [54]).

Furthermore, the choice of comparing parents from Italy and Portugal was made due to the interest in potentially addressing similarities and differences, strengths and weaknesses among these two systems [55]. Indeed, despite NICU policies across Europe, which may vary sensibly, NICUs of Southern European countries, such as Italy and Portugal, share the presence of higher restrictions on visiting policies by family and friends than Northern European countries [49]. Italy and Portugal also share values regarding the social function of family and other significant relationships [56,57]. Therefore, this comparison could provide valuable information to implement not only parent-tailored, but also country-specific interventions, while addressing the need for co-creating unified screening protocols and support interventions for NICU parents across Europe [58,59,60]. Accordingly, based on the abovementioned study objectives and the reported literature, the following hypothesis and research questions have been proposed and tested:

**Hypothesis 1** **(H1).** *Latent Classes: Parents of VPT infants can be grouped into classes according to perceived NICU stressors and sources of social support.*

*Research Question 1 (RQ1)—Cross-Country Validity*: Are the identified classes valid across Italian and Portuguese parents?

*Research question 2 (RQ2)—Predictors*: Does class membership vary according to sociodemographic factors?

## 2. Materials and Methods

### 2.1. Participants and Procedures

This is a comparative cross-sectional study gaining data from two larger research projects conducted in NICU settings in Portugal (between 1 July 2013 and 30 June 2014 [27,54,61]) and in Italy (from June 2023 to August 2024 [8,62,63]) in 8 III level NICUs (number of hospital staff working in NICUs varying from 5 to 8 physicians and 15–20 nurses, up to 18–26 physicians and 35–60 nurses, in larger and highly specialist centers).

The two research projects were approved by the Portuguese Data Protection Authority and the Ethics Committee for the Psychological Research of the University of Naples Federico II, respectively, and consent was also obtained by the hospitals where the studies were performed. The research was carried out in accordance with the Declaration of Helsinki and written informed consent was obtained from all participants.

Based on the convenience sampling method, parents of VPT infants hospitalized in NICUs in the Northern Health region of Portugal and in a Southern region of Italy were invited to fill out standardized questionnaires between the third and the fourth week after birth. Specifically, during the time frames for data collection, a total of 201 very preterm infants were born in Portuguese NICUs and 101 in the Italian NICU, respectively, corresponding to 165 (130 single pregnancies, 34 twin pregnancies and 1 triplet) and 76 families (54 single pregnancies, 20 twin pregnancies, 1 triplet and 1 quadruplet) that were considered potentially eligible. Inclusion criteria were as follows: 1. being a parent of a VPT infant hospitalized in the NICU at the time of data collection; 2. being ≥ 18 years old; 3. understanding Portuguese/Italian written form fluently, respectively. Exclusion criteria were the following: 1. Being a parent of an infant with congenital diseases or being a parent of an infant who was not likely to survive according to the physician’s opinion. 2. Being a parent yet not being present in the NICU during the infants’ hospitalization period.

Therefore, after excluding families whose infants were not hospitalized in the NICU at the time of the data collection, due to discharge, transfer to another hospital or death (Portugal = 2; Italy = 9), families with serious illness that precluded NICU visitation (e.g., severe chronic conditions; Portugal = 4; Italy = 2), families who were not present in the NICU during the baby’s hospitalization period (Portugal = 6; Italy = 3), and those who did not read neither Portuguese (*n* = 20) nor Italian (*n* = 1), overall, 133 families in Portugal and 61 families in Italy (both mothers and fathers) were found to be eligible and approached during the hospital stay (over the period of 15 to 22 days after birth) by a health professional (psychologist in Italy and neonatologist or nurse in Portugal), who was responsible for the study presentation and invitation.

Among the eligible participants who were informed about the project purpose, 49 Portuguese parents (13 mothers and 42 fathers) and 30 Italian parents (10 mothers and 20 fathers) refused to participate. Refusals by mothers were mainly justified by a lack of time to participate, while fathers who did not participate were mainly absent due to professional commitments.

Therefore, overall, 211 Portuguese parents (120/133 mothers, Response Rate = 90.2%; and 91/133 fathers, Response Rate = 68.4%) and 92 Italian parents (51/61 mothers *Response Rate* = 83.6%; and 41/61 fathers; *Response Rate* = 67.2%) agreed to participate. The surveys were completed by mothers and fathers separately in a silent and bright dedicated room, while a research psychologist (Italy) or a trained interviewer (Portugal) was present to answer any possible question raised by them.

The final dataset for the present study included 303 parents of VPT infants (92 Italian; 211 Portuguese). Considering power analysis, due to the a posteriori nature of the analysis, a thorough review of the literature on power analysis for MG-LCA was conducted to examine the adequacy of the sample size before performing MG-LCA. While some studies suggest that *N* = 30 would be sufficient for each class [64,65], others recommend a minimum sample size of 300 subjects [66,67,68,69,70]. Therefore, the number of participants included in the present study (*N* = 303) should be considered appropriate for carrying out MG-LCA according to the recommendations reported. Also, given the presence of some missing values, Little’s missing completely at random (MCAR) test [71] was used to assess whether the pattern of missingness was unrelated to the variables under study. A non-significant result in this test (*p* > 0.05) indicates that the data is missing completely at random. In our case, the test was non-significant (χ^2^ = 37.668, df = 26, *p* = 0.06), suggesting that the missing data in our dataset could be considered random. This result enabled us to handle the missing values using Full Information Maximum Likelihood (FIML) estimation [72].

### 2.2. Measures

Participants were asked to complete a survey including a *Background Information Sheet* for collecting sociodemographic factors along with two valid measurement tools, namely, the *Parental-Stressor-Scale-NICU* for the assessment of perceived NICU stressors (PSS: NICU [15]; Italian Version [73]; Portuguese Version [74]), and the *Multidimensional Scale of Perceived Social Support* for the assessment of sources of social support (MSPSS [75]; Italian Version [76]; Portuguese Version [77]).

Specifically, the *Background Information Sheet* was used to explore the following sociodemographic information: *Gender* (Women/Men); *Age* (in years); *Education level* (harmonized among the educational systems of the two countries and dichotomized as ≤12/>12 years of education); *Occupational status* and *Job Category* (classified according to the Portuguese Classification of Occupation [PCO] and the Italian classification of occupation from the Italian Institute of Statistics [ISTAT]); *Having other children* (having at least one other child = No/Yes); *Previous Abortion* (No/Yes); *Distance between home and the NICU* (Duration of trip from home to the NICU in minutes by car calculated using Google Maps (v.9.0.0)). Information about the *pPreterm delivery* (Extreme Preterm Delivery = No/Yes) and *Infant’s weight* (Extremely low birthweight = No/Yes) was also collected.

The *Parental Stressor Scale: Neonatal Intensive Care Unit* (PSS: NICU [16,73,74]) consists of 26 items divided into 3 subscales: *Sights & Sounds* (SISO; Portuguese sample α = 0.750; Italian sample α = 0.810), *Infant Look and Behaviour* (IBA; Portuguese sample α = 0.927; Italian sample α = 0.919) and *Parental Role Alterations* (PRA; Portuguese sample α = 0.854; Italian sample α = 0.880). Parents are asked to rate each item on a 5-point Likert scale ranging from 1 (not at all stressful) to 5 (extremely stressful) according to their perceived stress at the time of administration. Parents can also rate as 0 (not applicable) those items that are not related to their own experience during NICU hospitalization. An overall stress score is computed from the three subscales ranging from 1 to 5. As our focus of interest was related to parental experience across the two countries, we used the *Stress Occurrence Level* (SOL), thus including only experienced items [16].

The *Multidimensional Scale of Perceived Social Support* (MSPSS [75,76,77]) is a 12-item questionnaire on a Likert scale ranging from 1 (completely disagree) to 7 (completely agree). It is designed to measure the perceived social support from three different sources, namely: *Family* (Portuguese sample α = 0.905; Italian sample α = 0.950), *Friends* (Portuguese sample α = 0.960; Italian sample α = 0.942), and *Significant Others* (Portugues sample α = 0.819; Italian sample α = 0.930).

### 2.3. Data Analysis

Preliminarily, descriptive analysis (i.e., means/standard deviations, frequencies/percentages) was run for sociodemographics by using the software SPSS (version 29). Therefore, in order to test *Hypothesis 1* (H1; *Latent Classes*), Multigroup Latent Class Analysis (MG-LCA) was used. Specifically, MG-LCA was carried out using the glca package [78,79] of jamovi software v.2.6 [80]. In line with the objectives of this study, MG-LCA was chosen due to its ability to detect both universal and culture-specific types of response configurations when carrying out cross-country comparisons [38,81]. Moreover, in order to facilitate interpretation [82] and clearly display data, NICU stressors and social support scores were turned into 4 quartiles corresponding to the 25th, 50th, 75th and 100th quartile of the sample distribution. Specifically, perception of stress and social support were classified as 1 (25th quartile) = low, 2 (50th quartile) = medium/low, 3 (75th quartile) = medium/high, and 4 (100th quartile) = high. The stepwise approach [67] was used to select the number of classes that fits our data in the best way, starting from two classes and going ahead. Due to the spare nature of our data, we used the Bayesian information criterion (BIC [83]) and the Entropy score > 0.80 to define the best fitting data model [84].

Afterwards, in order to respond to *Research Question 1* (RQ1; *Cross-Country Validity),* measurement invariance was explored [84], while, in order to respond to *Research question 2* (RQ2; *Predictors*), multinomial logistic regression analysis was used to examine the associations among the sociodemographic variables and the membership to the emerged classes.

## 3. Results

Three hundred and three parents (51 mothers and 41 fathers from Italy; 120 mothers and 91 fathers from Portugal) were included in the study. Descriptive statistics are displayed in Table 1, and data showed that parents from Italy and Portugal did not statistically differ in sociodemographic data distribution, except for Age and Distance from NICU, with Italian parents being older and living more distant from NICU than parents from Portugal. Therefore, despite the differences in sample size, the two groups can be considered comparable.

**Figure 1:**
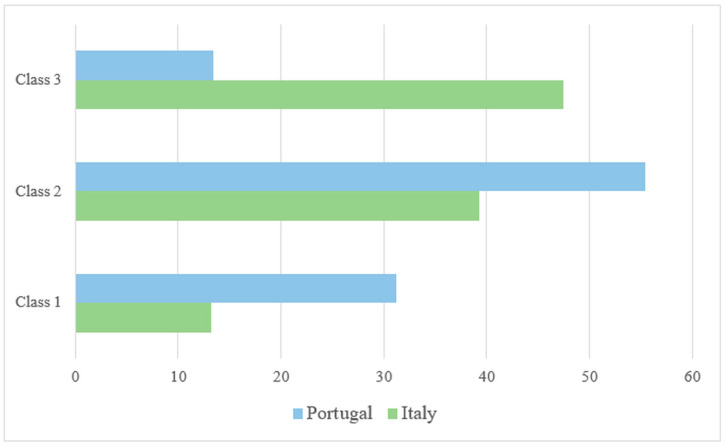
Class prevalence stratified by country.

Therefore, considering H1, data from Multigroup Latent Class Analysis (MG-LCA) confirmed that parents of VPT infants can be grouped into classes according to perceived NICU stressors and sources of social support. Specifically, data revealed that the 3-class model provided the best fit for the data (Table 2). In fact, this model had the lowest BIC score and the highest Entropy score. Plus, more than 5% of the subjects were present in each class, considering both the total sample (i.e., Class 1: 25.7%; Class 2: 50.5%; Class 3: 23.8%) and results across countries (Figure 1).

Therefore, considering RQ1, data also confirmed that the identified classes are valid across countries. Indeed, the BIC index in the model that accepts the measurement invariance (BIC = 3404) was better than the one that does not accept measurement invariance between groups (BIC = 3593).

**Figure 2:**
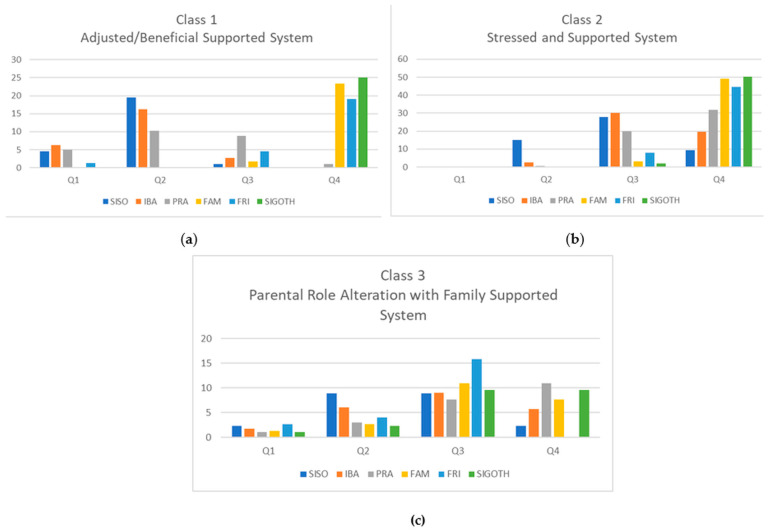
Rates of NICU stressors and sources of social support according to (**a**) Class 1, (**b**) Class 2, and (**c**) Class 3. Note. SISO = *Sights and Sounds stressor*; IBA = *Infant and Behaviour Appearance stressor*; PRA = *Parental Role Alteration stressor*; FAM = *Family Social Support*; FRI = *Friends Social Support*; SIGOTH = *Significant Others’ Social Support*.

Afterwards, in order to label the emerged cross-country classes, the crosstabs and the bar graphs showing the trends of the scores of NICU stressors and sources of social support for each class in both the Italian and the Portuguese samples were visually inspected (Figure 2). Class 1 accounted for 25.7% of the whole sample and was labelled as the *Adjusted*/*Beneficial Supported System* since it grouped parents reporting the lowest scores in all NICU stressors and high scores in all social support sources. Class 2 accounted for 50.5% of the whole sample and was labelled as *Stressed and Supported System* since it grouped parents reporting the highest scores for all NICU stressors (medium–high and high scores) yet also the highest scores for all the social support sources. Class 3 accounted for 23.8% of the whole sample and was labelled as *Parental Role Alterations with Family-Supported System* since it grouped parents reporting medium/low scores for two out of three NICU stressors, namely, “Sights and Sounds” and “Infant Behaviour and Appearance”, high and medium/high scores for the NICU stressor of “Parental Role Alterations”, along with medium/high scores for two out of three sources of support, namely, “Family” and “Significant Others”, with medium/low scores registered for “Friends” support.

Finally, considering RQ2, data from multinomial logistic regression revealed that class membership significantly varied according to specific sociodemographic factors, namely, Gender, Age, and having another child (Table 3). Specifically, men are less likely than women to belong to both Class 2, *Stressed and Supported System* (B [SE] = −0.718 [0.290], *p* = 0.013), and Class 3, *Parental Role Alterations with Family-Supported System* (B [SE] = −0.940 [0.377], *p* = 0.013), compared to Class 1, *Beneficial Supported System*. Moreover, younger age increased the likelihood of belonging to Class 2, *Stressed and Supported System* (B [SE] = −0.087 [0.041], *p* = 0.032).

An interaction effect among Age and Having Other Children was also found, with more advanced-age parents with another child/other children being more likely to belong to Class 3, *Parental Role Alterations with Family-Supported System,* compared to Class 1, *Beneficial Supported System* (B [SE] = 0.220 [0.076], *p* = 0.004).

## 4. Discussion

By applying a Person-Centered Approach, the present study aimed at deepening and comparing the experiences of parents of Very-Preterm-Infants (VPT) in NICUs from Italy and Portugal. This is with the main objective of customizing support interventions by identifying specific sub-groups (classes) of parents featured by different nuances of risks and resources (perceived levels of NICU stressors and sources of social support) and that could also require further attention according to Country of Belonging and sociodemographic specificities.

Findings provided evidence for three statistically valid and cross-country classes labelled as Class 1, *Adjusted/Beneficial-and-Supported-System*, Class 2, *Stressed-and-Supported-System*, and Class 3, *Parental-Role-Alteration-with-Family-Supported-System*. Specifically, Class 1, named *Adjusted*/*Beneficial and Supported System* (25.7% of the total sample), describes parents for whom all sources of informal social support are highly perceived and, conversely, perceived NICU stressors are low. This configuration could suggest the hypothesis that—for these parents—informal social support might have played a key protective role, fostering better adjustment processes, and even counteracting the inherently stressful experience within NICUs. These findings fully aligned with both variable-centered [85] and qualitative studies [22], which highlighted the importance of evaluating, activating, and enhancing informal social support networks within family-centered care (FCC) policies and practices to also reduce parental stress [44,46,47].

Furthermore, still considering Class 1, data also suggested that women (mothers) were less likely than men (fathers) to belong to this class (and more likely to belong to Class 2 and Class 3 than fathers). Thus, fathers could be more likely to be grouped within the *Adjusted*/*Beneficial-and-Supported-System.* This finding endorsed the literature underlining that fathers experiencing NICU may report lower levels of stress and better adjustment than mothers (e.g., [14,86]). Yet, notwithstanding these results, it seems necessary to also highlight the need to develop further research and interventions supporting mothers [25,87]. This would indeed provide both parents with the necessary resources to deal with the challenges of NICU experience.

In line with this, findings also highlighted that some categories of parents, such as mothers, may have specific needs that have not been fully addressed yet, or whose specific needs during NICU hospitalization might not be fulfilled only by informal social support. This is the case of parents belonging to Class 2, named *Stressed and Supported System*, which included about half of the parents from the whole sample (50.5%). Specifically, despite parents belonging to this class perceiving high levels of support from their social system, as typically experienced in Southern European countries [56,57], they still report overwhelming levels of stress related to NICU experience. This is particularly true for younger parents—who were found to be more likely to belong to this class. This result provides, then, further evidence to a still-open debate concerning age, featured by some studies stating that younger age represents a further risk factor exacerbating NICU-related stress in parents [54,85], while other research reporting advanced age as, instead, a key risk factor [88,89].

Notwithstanding, despite the age specificities, belonging to this class could also be the result of the mismatch between receiving informal social support and perceiving this as adequate. Indeed, being “close” to someone does not translate directly into being supportive effectively [22]. From this perspective, friends, significant others, and mainly family members (e.g., grandparents) could be willing to be involved actively to provide support [90], yet they could also be not able to understand the real challenges of the NICU experience, potentially even resulting in “unintentional” intrusiveness [31], or exacerbating stress by seeking constant information and assurance regarding infant medical condition. However, it should also be considered that—in some cases—informal support cannot be enough, even when adequate, due to the additional strains and challenges parents need to deal with, for example, due to financial, work-related, and social issues [62,91]. In line with the FCC [44,46,47], such conditions may require more structured and formal sources of support to be faced effectively, for example, interventions from governments, the community (e.g., financial aid, family policies), and the healthcare system (e.g., dedicated training delivered by nurses, psychological counselling and peer support groups [4]).

However, an even more tailored profile emerged when considering Class 3, named *Parental Role Alterations with Family-Supported System* (including 23.8% of the total sample). It was defined with a very specific pattern in which perceived stress related to parenting is combined with the presence of a familial supporting system. This class highlighted, therefore, that the alteration in parenting function could be the main stressor for a specific group of parents of VPT infants. These findings are in line with other quantitative studies in which *Parental Role Alterations* represented one of the most prominent stressors experienced by parents in NICUs [50,92], requiring tailored interventions (e.g., kangaroo care [93,94]). From this perspective, it is important to also highlight that it is now well-acknowledged that the active participation of parents in the care of newborns not only promotes the better adjustment of parents to NICU experience [17,18], yet it also contributes positively to the attachment process and growth development of newborns [4,19]. It is therefore essential to ensure and facilitate the contact between parents and hospitalized infants and—whenever possible—the execution of care duties.

Moreover, findings on Class 3 also shed light on an even more specific group of parents, those of an advanced-age and having other children, who are more likely to belong to this class, and who may struggle due to the unique challenges to balance parenting “in and out” of the NICU. Also, this group of parents seems to feel overall lower levels of support, and this mainly derives from the family system rather than by the extended network (e.g., friends), thus probably receiving practical and instrumental support such as help in caring for other children, assisting with household chores, and providing transportation from-to-NICUs.

Though, overall data on class membership highlighted that, on one side, NICU stressors can be perceived as low, overwhelming or in a very specific nuance (e.g., Parental Role Alteration), whereas, on the one other side, informal social support is fairly widely perceived by all parents of very preterm infants in NICUs (i.e., medium-to-high levels were found predominant across the three classes). Thus, data suggested that, despite this source of support being fundamental, it could also have a limited protective role, particularly for specific groups (e.g., mothers, advanced-age parents with other children). This will need further research and interventions able to capture their additional and specific needs, as well as to identify further protective factors (e.g., individual and personality characteristics; sources of formal support) able to help them to deal with this extreme and emotionally demanding experience.

Regarding the cross-country comparison, our study highlighted that the three classes were meaningful and statistically valid both in Italy and Portugal, and so it would potentially be useful to implement shared policies, practices, and interventions among these two countries. However, data also revealed that parental distribution was different across countries. Specifically, Portuguese parents were mainly grouped in Class 1, *Adjusted/Beneficial-and-Supported-System*, which is fair to hypothesize as including parents who are better adjusted to NICU experience, and in Class 2, *Stressed and Supported System*. This indicates the possible need for more formal, structured, and professional support to deal with the burden of NICU stressors. Differently, Italian parents were mainly grouped in Class 3, *Parental Role Alterations with Family-Supported System,* thus struggling to a greater extent with perceived stress linked to feelings of inadequacy in caring for their own infant, feeling less supported, and relying to a greater extent on the family system. This could, however, be also linked to data showing that older parents having other children were more likely to belong to Class 3. Indeed, Italian parents were found to be older and, despite not being statistically significant, reported having other children to a greater extent than the Portuguese ones. In line with this, Italy is also one of the European countries with the highest maternal age at the time of the first childbirth [95] and advanced-age pregnancies are well-recognized to be at higher risk, often coming after previous fertility treatment failures [96] and/or several perinatal complications [97]. These factors then could lead advanced-age parents to reach NICU admission with an additional burden related to their previous path.

Moreover, cross-country differences in class membership could also reflect previous findings showing that both Italian parents and nurses in NICUs experienced lower quality of FCC as compared to other European countries [51]. This is potentially due to several barriers affecting FCC implementation, including staff shortage, lack of emotional training for nurses, scarce financial resources [98,99] and scantiness of structured organizational models [100]. From this perspective, the lack of formal support might also limit the beneficial effect of the informal social support system, indicating the need for the structural implementation of spaces and healthcare staff able to detect and address these specific needs.

However, above all, these findings shed light on similarities and differences in NICU experiences across two similar European countries, suggesting the need for the implementation of shared yet personalized FCC practices according to different parental needs and social and healthcare contexts.

### 4.1. Implications and Recommendations

Based on the person-centered approach, the present study provided researchers and practitioners with information on three cross-country valid classes that can be used to group parents of very preterm infants. They share the NICU experience, yet they significantly differ in the risk profiles, suggesting tailored implications for research and practice.

Firstly, to the best of our knowledge, this is the first study using a person-centered approach to simultaneously address risks (NICU stressors) and resources (sources of social support), thus highlighting the potential of this approach in differentiating and understanding—to a greater extent—the specific experiences of parents of VPT infants (considered as a high-psychological-risk population). This, indeed, can inform the definition of customized actions in the healthcare setting based on a structured application of the FCC Model, which works based on evidence collected within a specific setting.

Specifically, the identification of Class 1, *Adjusted*/*Beneficial-and-Supported-System*, implies that parenting VPT infants in NICUs should not be considered inherently overwhelming, since some parents can deal with it effectively, particularly when the support system is considered adequate. For these parents, careful monitoring (from the healthcare staff—mainly nurses and psychologists working at NICUs) is recommended, ensuring the maintenance of balance and adjustment not only during NICU hospitalization but also at transition home with their own infant. From this perspective, although the preparation of parents for discharge should be considered by healthcare staff from admission to NICU [101], the inherent uncertainty of the condition could hinder this process. Discharge could, therefore, represent a further challenge, potentially requiring different support interventions for parents.

However, the identification of Class 2, *Stressed-and-Supported-System,* implies that, only for some parents (more likely mothers), this experience represents a great challenge that can exceed their own resources. In such cases, informal support can be “not enough”, requiring the activation of more formal, structured and professional sources of support.

Similarly, yet with even more specific features, the identification of Class 3, *Parental-Role-Alteration-with-Family-Supported-System,* implies that for some parents’ (more likely older and having other children) interventions should mainly focus on fostering parent–infant relationships. Moreover, decentralizing the sources of support and expanding the support network beyond the family—with a higher commitment from professionals and other significant relational resources—could also be recommended in such cases.

However, overall, the identification of Class 2 and Class 3 means that specific implications and recommendations must be considered for the effective implementation of the FCC Model, for which the experience of each family member within NICUs requires a structured assessment to help target their unique needs effectively [45]. In other words, to clearly identify parents belonging to Class 2 and Class 3, psychosocial screening procedures are highly recommended [55,59].

Nevertheless, these procedures should take into account parents and their cultural and social context [102,103]. From this perspective, the detection of statistically valid classes across Italy and Portugal, while also accounting for country specificities, could not only raise awareness of policymakers on similarities, differences and disparities that are still present within the European contexts in terms of NICU policies, practices, as well as parents’ experiences. Yet, this information could also encourage the development of further cross-country research and tailored interventions that aim at reducing disparities. From this perspective, data on the higher presence of Portuguese parents in Classes 1 and 2, and the higher presence of Italian parents in Class 3, suggest the need to increase the efforts to co-create and implement shared practices and interventions at the European level to guarantee the same access and implementation of FCC practices.

In the same direction, more emphasis should be placed on how health systems and policymakers can practically support the vulnerable parent groups identified in the study. Indeed, findings on the role of specific sociodemographic risk factors call for policymakers and healthcare professionals to develop actions targeting specific groups of parents (i.e., mothers, advanced-age parents with other children). They were indeed found to be at higher risk of experiencing stress, parenting difficulties and a lack of beneficial social support system, requiring tailored attention.

Furthermore, it is noteworthy to advise that, once specific needs have been identified, NICU healthcare staff (neonatologists, nurses, psychologists) should plan and implement interventions accordingly. These could include integrating peer-to-peer support groups (e.g., [104]) and psychological support programs (e.g., [105]), which, however, need to involve members of NICU staff throughout the intervention (from its design) and to also ensure equal access to different subgroups of parents of VPT infants (e.g., including mothers and fathers and people from different cultural and socio-economic backgrounds).

Moreover, since the NICU staff and, in particular, nurses, are at the forefront of the relationship with parents, and they are essential to foster parent–infant relationships, further implications and recommendations may be derived from the present study to support parents belonging to the three emerged classes. Specifically, it is recommended that NICU medical staff and psychologists participate in courses and lifelong learning activities. This will allow them to be properly trained to implement actions (in daily work life), programs and interventions to support parents effectively both during infant hospitalization and after discharge (e.g., [4]).

### 4.2. Study Limitations

Notwithstanding these important implications, some limitations should be considered. Firstly, the cross-sectional design of the study precludes any conclusions about causality and weakens the interpretation of the interplay of risks and resources among parents of VPT infants. Future research could be developed to collect longitudinal follow-up data to test the replicability of our findings and to improve the applicability of the results, but also to potentially verify the effectiveness of evidence-based interventions developed upon our findings and, accordingly, implemented differently across the three subgroups of parents.

Secondly, the data were collected from only one source, namely, self-report measures completed by parents, thus limiting the possibility to further deepen parents’ nuances of experience and increasing the risk of answers influenced by social desirability bias. Therefore, future research could also include a wider range of sources of data and could also be designed to explore the perspective of other key actors (e.g., members of the informal support network, such as grandparents, and the healthcare staff).

Moreover, given the cross-country comparison, potential cultural differences affecting parental responses to questionnaires should also be addressed [106]. Nevertheless, it should also be considered that the shared geographical area (Southern Europe), in which rather similar patterns of responses are registered [107,108], and the adoption of measurement tools that have been statistically validated in Italy and Portugal, respectively, could have limited the results to a lesser extent than if North/Western European countries had been considered in the study.

Furthermore, despite the overall sample being representative of parents of VPT infants and large enough to respond to hypothesis/research questions, the Portuguese (larger) and the Italian (smaller) samples were different in size, and mothers were—in both countries—more represented than fathers, thus limiting the generalizability of research results. Therefore, further studies on larger/nationally balanced samples—always involving both mothers and fathers within the parenting couple in research design—could be developed to strengthen the generalizability of these results. However, differences across sample sizes are mainly due to the differences in study design between the two larger projects conducted in Italy and Portugal. In particular, the broader Italian project included parents of very preterm infants but was not limited to them, as it also included other preterm categories.

In line with this, another aspect potentially limiting the cross-country comparison concerned the different times of data collection between the two countries. Nevertheless, it is important to note that the variables examined in the study—namely, parents’ subjective experiences of the stressors associated with the extreme experience of having a very preterm infant in the NICU, between life and death, and their perception of sources of informal support—are not likely to be affected by the years. Indeed, while formal and structural support may have been subject to changes over the years due to efforts to implement increasingly effective support practices and policies in the healthcare system, the variables explored in this study pertain to the parents’ personal experiences and are unique, somewhat time-invariant, and thus comparable across two sub-groups of the same population with data collected at different years. Still, future cross-country studies adopting a person-centered approach and addressing all these limitations could significantly enrich research and underpin the development of interventions fostering wellbeing in NICU units. In this direction, future research should account for potential organizational differences between NICUs across European countries and should also include further variables potentially playing a key role in influencing NICU experiences of parents of VPT infants (e.g., personality characteristics, structured sources of formal support systems).

## 5. Conclusions

In conclusion, the experience of parents of VPT infants in NICUs is inherently challenging, yet assessing, identifying, and monitoring specific risk profiles featured by the unique nuances of stressors and sources of support while accounting for further factors (Country of Belonging, Gender, Age, Having another child) can foster the customization of interventions to provide parents with the necessary resources for adjusting to this extremely demanding experience and implementing FCC practice effectively.

## Figures and Tables

**Table 1:** Descriptive statistics of sociodemographic data of the whole sample and stratified by country.

	Total Sample (*N* = 303)	Portuguese Sample (*N* = 211)	Italian Sample (*N* = 92)	*p* Values
**Gender** *N* (%)				
Women	171 (56.4)	120 (56.9)	51 (55.4)	
Men	132 (43.6)	91 (43.1)	41 (44.6)	0.817
**Age** *M* (SD)	32.8 (5.65)	32.14 (5.37)	34.23 (6.01)	0.003 **
**Education level** *N* (%)				
>12	117 (38.6)	79 (37.4)	38 (41.3)	
≤12	186 (61.4)	132 (62.6)	54 (58.7)	0.525
**Occupational status** *N* (%)				
Employed	240 (79.2)	166 (78.7)	74 (80.4)	
Not employed	63 (20.8)	45 (21.3)	18 (19.6)	0.728
**Job Category**				
Upper white collar	113 (37.3)	81 (38.4)	32 (34.8)	
Lower white collar	73 (24.1)	57 (27.0)	16 (17.4)	
Blue collar	77 (25.4)	53 (25.1)	24 (26.1)	
Missing	40 (13.2)	20 (9.5)	20 (21.7)	0.766
**Having Other Children** *N* (%)				
Yes	98 (32.3)	57 (27.0)	41 (44.6)	
No	206 (60.1)	154 (73.0)	51 (55.4)	0.277
**Previous Abortion** *N* (%)				
Yes	89 (29.4)	57 (27.0)	32 (34.8)	
No	214 (70.6)	154 (73.0)	60 (65.2)	0.172
**Distance from the NICU** *M* (SD)	27.94 (22.11)	19.79 (17.76)	39.04 (24.69)	0.000 ***
Missing	88	87	1	
**Extreme preterm delivery** ^a^ *N* (%)				
Yes	80 (26.4)	45 (21.3)	35 (38.0)	
No	223 (73.6)	166 (78.7)	57 (62.0)	0.143
**Extremely low birthweight** ^b^ *N* (%)				
Yes	94 (31.0)	63 (29.9)	31 (33.7)	
No	209 (69.0)	148 (70.1)	61 (66.3)	0.507

Note. ^a^ Delivery > 28 gestational weeks; ^b^ birthweight < 1000 g. Differences are calculated by Student’s *t* test (*M* = Means/SD = Standard Deviations) or Chi-square test (*N* = Number/% = Percentages). ** *p* < 0.01; *** *p* < 0.001.

**Table 2:** Model fit indices for number of class solutions.

Class	Log-Likelihood	AIC	CAIC	BIC	Entropy	Df	G^2^
2	−1608	3291	3470	3432	0.766	264	846
**3**	**−1536**	**3189**	**3462**	**3404**	**0.822**	**244**	**703**
4	−1501	3158	3525	3447	0.791	224	632

**Table 3:** Multinomial logistic regression of sociodemographic variables predicting Class 2 and Class 3 membership with the Beneficial Supported System (Class 1) as reference.

	OR [95% CI] ^a^	
	Class 2 ^b^	Class 3 ^c^
**Gender**		
Women	1	1
Men	488 * [0.276, 0.861]	0.391 * [0.186, 0.819]
**Age** (in years)	0.917 * [0.847, 0.993]	0.947 [0.846, 1.061]
Age * Having Other children		
Having Other children—No	1	1
Having Other children—Yes	1.063 [0.943, 1.198]	1.246 * [1.074, 1.445]

Note: Only significant results are displayed. ^a^ Reference class: Adjusted/Beneficial Supported System (Class 1); ^b^ Class 2: Stressed and Supported System; ^c^ Class 3: Parental Role Alterations with Family-Supported System. * *p* < 0.05.

## Data Availability

The data presented in this study are available on request from the corresponding author.

## Abbreviations

The following abbreviations are used in this manuscript:

IBA	Infant and Behaviour Appearance
FAM	Family Social Support
FCC	Family-Centered Care Model
FRI	Friend Social Support
MG-LCA	Multigroup Latent Class Analysis
NICU	Neonatal Intensive Care Unit
PRA	Parental Role Alteration
SIGOTH	Significant Others’ Social Support
SISO	Sights and Sounds
VPT	Very Preterm
